# Research on the Improvement of Semi-Global Matching Algorithm for Binocular Vision Based on Lunar Surface Environment

**DOI:** 10.3390/s23156901

**Published:** 2023-08-03

**Authors:** Ying-Qing Guo, Mengjiao Gu, Zhao-Dong Xu

**Affiliations:** 1 College of Mechanical and Electronic Engineering, Nanjing Forestry University, Nanjing 210037, China; gumengjiao@njfu.edu.cn; 2 China-Pakistan Belt and Road Joint Laboratory on Smart Disaster Prevention of Major Infrastructures, Southeast University, Nanjing 210096, China

**Keywords:** stereo vision, Semi-Global Matching algorithm, census transformation, adaptive window

## Abstract

The low light conditions, abundant dust, and rocky terrain on the lunar surface pose challenges for scientific research. To effectively perceive the surrounding environment, lunar rovers are equipped with binocular cameras. In this paper, with the aim of accurately detect obstacles on the lunar surface under complex conditions, an Improved Semi-Global Matching (I-SGM) algorithm for the binocular cameras is proposed. The proposed method first carries out a cost calculation based on the improved Census transform and an adaptive window based on a connected component. Then, cost aggregation is performed using cross-based cost aggregation in the AD-Census algorithm and the initial disparity of the image is calculated via the Winner-Takes-All (WTA) strategy. Finally, disparity optimization is performed using left–right consistency detection and disparity padding. Utilizing standard test image pairs provided by the Middleburry website, the results of the test reveal that the algorithm can effectively improve the matching accuracy of the SGM algorithm, while reducing the running time of the program and enhancing noise immunity. Furthermore, when applying the I-SGM algorithm to the simulated lunar environment, the results show that the I-SGM algorithm is applicable in dim conditions on the lunar surface and can better help a lunar rover to detect obstacles during its travel.

## 1. Introduction

The lunar surface is topographically complex due to the presence of numerous rocks and craters, which present significant challenges for the scientific investigations carried out by lunar rovers. These lunar rovers must efficiently undertake crucial tasks, including obstacle detection, path planning, and obstacle avoidance. However, the low level of illumination on the lunar surface leads to poor contrast in the captured images, which causes a loss of image details, thereby hindering further image processing [1,2]. Therefore, the lunar rover must carefully select its sensor. Binocular cameras are most cost-effective sensors and can provide rich and high-dimensional semantic information for obstacle detection tasks, while having a lower power consumption [3,4,5,6,7] and better assisting the lunar rover with its exploration mission.

Binocular vision [8,9] is a significant subfield in machine vision, where 2D planar image data are acquired by two cameras and extended to a 3D scene using computer vision processing. Binocular vision relies on stereo matching to determine disparity by matching the equivalent pixel locations in an image pair. The precision of the final reconstruction is directly proportional to the quality of the stereo matching result. With the gradual development of research in this field, stereo matching is being widely used in 3D reconstruction [10,11], industrial inspection [12,13,14,15], UAV [16,17], remote sensing [18,19], and intelligent control of robots [20,21,22].

Scharstein et al. [23] divided stereo matching algorithms into two categories: local stereo algorithms and global stereo algorithms. The local stereo matching algorithm determines disparity by comparing image blocks of the same size in the left and right views. Although the local stereo matching algorithm has a low computational complexity, it has poor matching effects in low texture areas, repeated texture areas, disparity discontinuities, and occlusion areas. It is also prone to mismatching. The global stereo matching algorithm calculates disparity by constructing a global energy function and minimizing it. Although the global stereo matching algorithm has a high matching accuracy, it is computationally intensive, resulting in a low algorithm rate, so it is not suitable to use the global stereo matching algorithm in instances with high real-time requirements, and cannot be implemented in hardware. Based on the above problems, Hirschmuller [24] proposed a Semi-Global Matching (SGM) algorithm, which can meet the requirements of both the high accuracy and efficiency of the algorithm. The SGM algorithm has become a major part of scholars’ research in recent years.

To improve the robustness of aggregation, many researchers have fused SGM algorithms with other algorithms. Guo et al. [25] proposed a modified SGM algorithm based on the fast Census transform. Zhang et al. [26] proposed a PatchMatch Semi-Global Matching (PMSGM) algorithm that significantly reduces the number of candidate disparity through a PatchMatch spatial propagation scheme. Pan et al. [27] proposed an improved Census transform algorithm based on the sequence template. Zhang et al. [28] used the Census transform and adaptive window to calculate cost simultaneously, and determined the size and shape of the cost calculation window by judging the gradient size of the matching points. The SGM algorithm often has similar texture and fringe artifacts due to the lack of interaction between adjacent scanlines. In response, Liu et al. [29] propose a method of layering and precise positioning of the disparity search space using SGM pyramids and local invariant features to improve computational efficiency, reduce memory occupation, and reduce the influence of similar textures. Bu et al. [30] introduced a local edge-aware filtering method in SGM to enhance the interaction of adjacent scanlines and avoid fringe artifacts. Deng et al. [31] combined multi-path cost aggregation and cross-scale cost aggregation (CSCA) to propose an SGM-MSIF algorithm based on multi-scale information fusion (MSIF). In order to achieve the high real-time performance of SGM algorithms, many scholars have combined SGM algorithms with FPGA. Sawant et al. [32] show the design and implementation of a stereo-vision system, which is based on an FPGA implementation of More Global Matching (MGM). MGM is a variant of SGM. To reduce the effect of noise on matching accuracy, researchers usually combine SGM algorithms with image filtering methods, such as derivative filtering [33,34], bilateral filtering [35], and median filtering [36].

Although all the above algorithms achieve better accuracy, they still have the problems of excessive computation and time consumption in practical situations. In the case of the same texture large area, the matching effect and accuracy obtained by these algorithms are also less satisfactory and poor. To address the above problems, this paper proposes the improved SGM (I-SGM) algorithm, which combines an adaptive window based on connected components and an improved Census algorithm. Firstly, the Census algorithm is used in the cost calculation instead of the original mutual information-based cost calculation. The improved Census algorithm reduces the influence of the window center pixel value on the Census algorithm, which effectively reduces the influence of noise on the algorithm and improves noise immunity. Second, since the matching window in the traditional Census algorithm is a fixed rectangular window, which will affect the matching efficiency of the algorithm, this paper proposes an adaptive window based on the connected components, which can be adaptive to obtain the best matching window for different regions of the image and reduce the running time of the algorithm. Finally, the accuracy of the algorithm is improved by using the cross-based cost aggregation in the cost aggregation stage. In order to verify the effectiveness of the I-SGM algorithm, the experimental analysis of the image pairs from the existing dataset is conducted. The experimental results show that the I-SGM algorithm can not only improve the matching speed and accuracy, but also has certain noise immunity.

## 2. Focused Problems

### 2.1. Semi-Global Matching Algorithm

The Semi-Global Matching algorithm uses the framework of the global algorithm to search through all regions of the image and a global optimization theory approach to estimate disparity [24]. The main principle of the algorithm is to establish a global energy function and use the results of aggregating multiple 1D paths to calculate the optimal solution for minimizing the energy function. The algorithm is divided into the following steps.

1. Pixelwise Matching Cost Calculation:

Mutual information (MI) [37] is used to characterize the magnitude of the amount of information shared by two images, which can be used as a measure of similarity between two images. This method is more robust to illumination changes as a pixel similarity metric and is an excellent cost function. The definition of MI is:

(1) $$MI_{I_{1},I_{2}}=\sum_{p}mi_{I_{1},I_{2}}\left(I_{1p},I_{2p}\right)$$

(2) $$mi_{I_{1},I_{2}}\left(i,k\right)=h_{I_{1}}\left(i\right)+h_{I_{2}}\left(k\right)-h_{I_{1},I_{2}}\left(i,k\right)$$

where *I*_1_ is the reference image; *I*_2_ is the matched image; **p** is a pixel point; *I*_1**p**_ and *I*_2**p**_ are the grayscale values of the left and right images, respectively; $mi_{I_{1},I_{2}}$ is the mutual information calculated for a corresponding point of *I*_1_ and *I*_2_; *i* and *k* are the two grayscales of the data items involved in the calculation; $h_{I_{1}}$ and $h_{I_{2}}$ are the entropy of *I*_1_ and *I*_2_, respectively; and $h_{I_{1},I_{2}}$ is the joint entropy of the two images.

The matching cost CMI for a point **p** based on mutual information is:

(3) $$C_{MI}\left(p,d\right)=-mi_{I_{b},f_{D}\left(I_{m}\right)}\left(I_{bp},I_{mq}\right)$$

(4) $$q=e_{bm}\left(p,d\right)$$

where *f_D_*(*I_m_*) is the base image, *I_b_* is the matched image, *I_b_***_p_** is the intensity of the base image pixel **p** and *I_m_***_q_** is the suspected correspondence of the matched image, and **q** denotes the eponymous point of pixel **p**.

2. Cost Aggregation

The matching cost in the previous section is susceptible to mismatches and noisy points. Therefore, in order to add smoothness constraints in the cost aggregation, the penalty function is considered to be constructed using the neighboring disparities. A global energy function *E*(*D*) is designed and used to integrate the matching cost and smoothness constraints.

(5) $$E(D)=\sum_{p}\left(C(p,D_{p})+\sum_{q\in N_{p}}P_{1}T\left[\left|D_{p}-D_{q}\right|=1\right]+\sum_{q\in N_{p}}P_{2}T\left[\left|D_{p}-D_{q}\right|>1\right]\right)$$

where *N***_p_** is the matching window and *P*_1_ and *P*_2_ are the penalty factors. The first term of the formula is the data term, which is the sum of the matching cost of all pixels under the disparity map *D*. The second and third terms are smoothing terms. The second term represents a penalty for cases where the difference in disparity between pixel **p** and its neighborhood pixel **q** is one pixel, adding a constant penalty value *P*_1_. The third term indicates that if the disparity between pixel **p** and neighboring pixel **q** is greater than one pixel, a constant penalty value *P*_2_ is added, and *P*_2_ > *P*_1_.

In order to convert the global energy function value to a fixed function energy value, path cost aggregation is performed. The 1D aggregation of the matching cost on all paths around a pixel yields the path generation value under the path, and the sum of all path generation values is the matching generation value after aggregation for that pixel. The path cost of a pixel **p** along a particular path **r** is calculated as shown in Equation (6).

(6) $$\begin{array}{cc} L_{r}(p,d)= & C(p,d)+\min(L_{r}(p-r,d),\\ & L_{r}(p-r,d-1)+P_{1},\\ & L_{r}(p-r,d+1)+P_{1},\\ & \underset{i}{\min}L_{r}(p-r,i)+P_{2})-\underset{k}{\min}L_{r}(p-r,k) \end{array}$$

where **r** refers to the path direction of pixel **p** and *d* is the disparity value of pixel **p**. *L*_**r**_(**p**,*d*) is the cost of matching when pixel **p** has value *d* and path direction **r**. **p** − **r** indicates the last matched pixel point when the path direction is **r**.

3. Disparity Computation

The Winners-Takes-All algorithm (WTA) [38] is usually used to calculate the disparity. The disparity corresponding to the smallest generation value is chosen as the optimal disparity among all the generation values for a given pixel. If scene accuracy is required, the disparity is calculated by fitting a quadratic curve to the neighboring costs at the neighboring disparity. A left–right consistency check can also be used to determine if there are masked areas and mismatched areas.

4. Disparity Refinement

The disparity image resulting from the above steps may still contain errors or invalid areas. These problems can be solved via the post-processing of the disparity image. If there are outliers in the disparity image, a method of removing peak disparity can be used. To solve the problem of disparity discontinuities, leading to boundary errors, intensity-consistent disparity selection can be performed on disparity images. When the disparity map has holes, it is necessary to obtain a dense disparity map by interpolation.

### 2.2. Census Algorithm

The basic principle of the Census algorithm is to create a window centered on a reference pixel point and use this window as a filter to traverse the matched image one by one. The entire filter makes the neighboring pixels form a binary string, and comparing the distance between the two strings is the value of the match between the reference pixel point and the matching pixel point [39].

The Census transformation formula is as follows:

(7) $$\varphi (I(p),I(q))=\left\{\begin{array}{l} 1,I(p)>I(q)\\ 0,I(p)<I(q) \end{array}\right.$$

where *I*(**p**) is the gray value of the pixel at the center point of the window, and *I*(**q**) is the gray value of the remaining points except the center point.

After setting the fixed window, all 1s or 0s are shot in the window into a string of bits. By comparing the bit strings of a pixel in two figures, the Hamming distance similarity measure can be used to derive the cost of matching as follows:

(8) $$h(p,d)=Hamming\left(C_{TL}(p),C_{TR}(p,d)\right)$$

Although the Census transform can accurately correct the grayscale deviation caused by uneven lighting, the Census transformation only uses a 0/1-bit byte string to represent the grayscale difference between two pixel points, making the feature descriptor too single. Additionally, when the grayscale changes sharply in the adjacent window, or there is the same repeat region, the matching cost obtained by the Census transformation does not reflect the similarity of the image well.

## 3. Improved Semi-Global Matching (I-SGM) Algorithm

**Figure 1:**
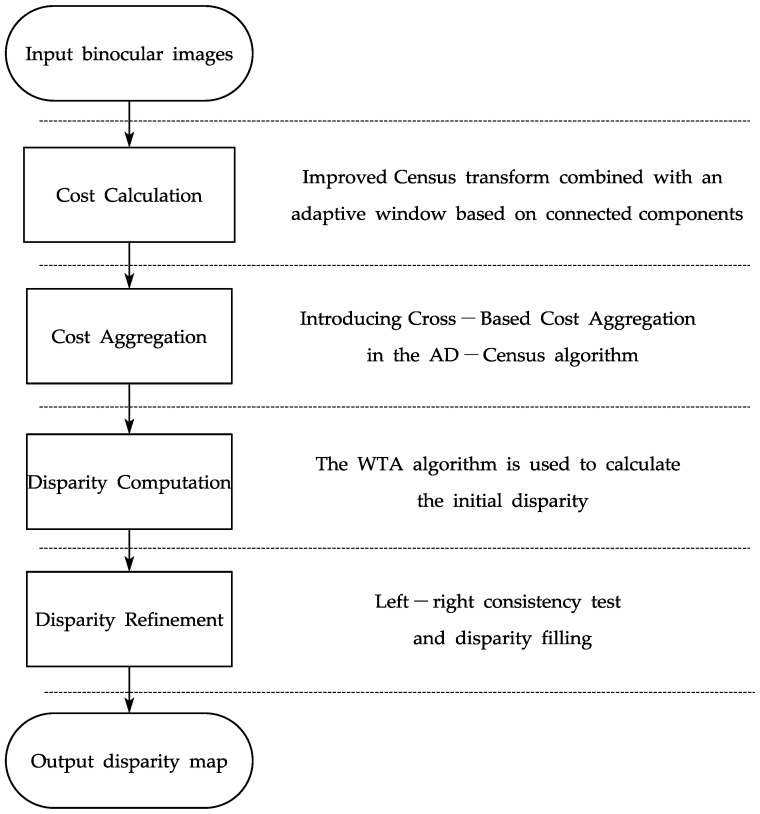
Flowchart of the I-SGM algorithm.

To improve the matching speed and accuracy of the algorithm, the I-SGM algorithm is proposed. For cost calculation, an improved Census algorithm is employed. The Census transform’s reference pixel is reset, and the adaptive transformation window based on the connected components is added into the improved Census algorithm. Cross-based cost aggregation is used in the cost aggregation. The WTA algorithm is used to calculate the initial disparity in the disparity computation. The initial disparity is then optimized using the left–right consistency test and disparity filling to obtain the final disparity map. The flow chart of the I-SGM algorithm is shown in Figure 1.

### 3.1. Improved Census Algorithm

**Figure 2:**
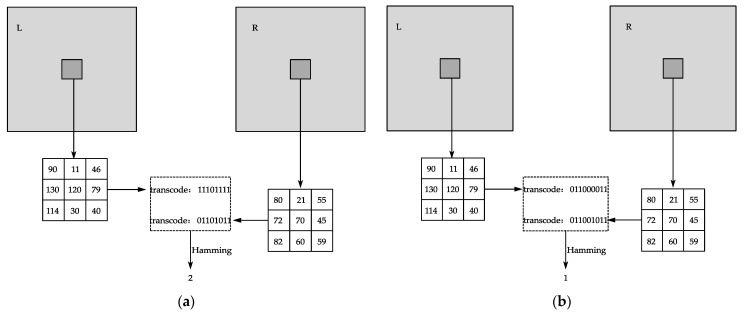
Census transformation process: (**a**) original Census algorithm; (**b**) improved Census algorithm.

The traditional Census transform relies too much on the central pixel point. When the central pixel point is disturbed by noise, the binary bit string obtained by the Census transform will significantly change, which is not conducive to maintaining the stability of algorithm performance [40]. In response, we redetermined the greyscale value of the reference pixel in order to improve its sensitivity to noise. In this paper, the greyscale values of neighboring pixels within the window are sorted, the maximum and minimum values are removed, and the average of the remaining pixel greyscale values is used as the greyscale value of the reference pixel. The improved equation is shown as below:

(9) $$\varphi (I(p),I(q))=\left\{\begin{array}{l} 1,I(p)>I(q)\\ 0,I(p)<I(q) \end{array}\right.$$

where *I*(**p**) is the average grayscale value of the remaining pixels in the window after removing the maximum and minimum values, and *I*(**q**) is the grayscale value of each point in the window. The process of Census transformation is shown in Figure 2.

As shown in Figure 2a, under the 3 × 3 window of the conventional Census algorithm, the reference pixel is the center pixel, whose value is 120, and the binary string after Census transformation is 11101111. After the above improved calculation, the reference pixel value is 74 and the binary string after improved Census transformation is 011000011, as shown in Figure 2b. The improved Census algorithm effectively suppresses the luminance difference by eliminating the extreme values, increases the coding length, weakens the influence of the central pixel point, and reduces the mismatch rate generated when the central pixel point is mutated.

### 3.2. Adaptive Window Based on Connected Components

The choice of window size is a key issue in the cost calculation. To ensure a more accurate disparity estimate without affecting calculation speed, the window scale must also meet the requirements of including sufficient intensity changes to obtain reliable matching and avoiding the influence of interference information. This study proposes a method to select a suitable window based on image connected component information from adaptation. A connected component is a collection of pixels made up of neighboring pixels with the same pixel value. Each connected component in the image is found and marked, and the size of the Census transformation window is determined by the size of the connected component inside the window. The process of the adaptive window is specified as follows:

(1) Set the initial window size to *I* × *I*, set the maximum window size to *M* × *M*, and set the initial number of connected components to *T*.
(2) Compare the initial window size *I* with the set maximum value *M*. If *I* < *M*, the number of connected components *X* of the same value as the center pixel is calculated; if not, then output the resulting window size *I* × *I*.
(3) Compare the number of connected components *X* of the same value as the center pixel with the initial number of connected components *T*. If *X* < *T*, output the resulting window size *I* × *I*; otherwise, *M* = *M* + 2, *T* = *T* + 1, and then skip to step (2).
(4) Output the matching window size *I* × *I*.

**Figure 3:**
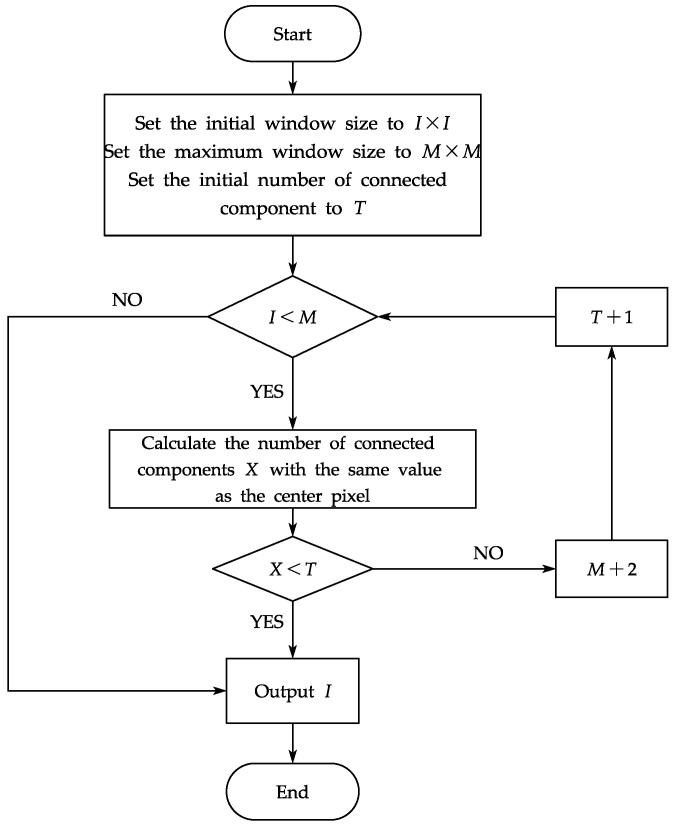
The specific flow of the adaptive window algorithm.

The specific flow of the adaptive window algorithm is shown in Figure 3.

### 3.3. Cross-Based Cost Aggregation

The cost calculation only considers the correlation between neighborhood pixels, so for the subsequent calculation of disparity, the correlation between pixels needs to be described in more detail via cost aggregation. This paper introduces improved cross-based cost aggregation in the AD-Census algorithm [41]. Cross-based cost aggregation assumes that pixels of similar colors in the neighborhood have similar disparity values. If the disparity values of the pixels participating in the aggregation process are close, the reliability of the aggregation will be higher. Cross-based cost aggregation is to build a cross-arm with the center pixel as the origin. The length of the cross arm is dynamic, and its arm length stops when it encounters a large difference in color or brightness values from the center pixel.

The specific algorithm for the cross domain is shown in Equation (10):

(10) $$\left\{\begin{array}{l} D_{c}\left(p_{1},p\right)<\tau_{1}\;and\;D_{c}\left(p_{1},p+(1,0)\right)<\tau_{1}\\ D_{s}\left(p_{1},p\right)<L_{1}\\ D_{c}\left(p_{1},p\right)<\tau_{2}\;if\;L_{2}<D_{s}\left(p_{1},p\right)<L_{1} \end{array}\right.$$

where **p** is the pixel to be matched; **p**_1_ is the new pixel; *D*_c_ (**p**_1_, **p**) is the difference between the color values of **p**_1_ and **p**; *D*_s_ (**p**_1_, **p**) is the arm length between **p**_1_ and **p**; *L*_1_, *L*_2_ are the thresholds for arm length; and *τ*_1_, *τ*_2_ are the thresholds for the difference between color values.

In this paper, the threshold value is reset. *D*_c_ (**p**_1_, **p**) is set to the difference between the gray values of **p**_1_ and **p**; *τ*_1_, *τ*_2_ are set as the thresholds for the difference between the gray values.

Cross-based growth (using horizontal as an example) must satisfy the following rules. If any of these rules are violated, the window stops growing.

(1) The gray difference between the point **p** to be matched and the new pixel **p**_1_, and the gray difference between the pixel to be added and the next pixel in this direction, are less than *τ*_1_.
(2) The arm length *D*_s_ (**p**_1_, **p**) of the point to be matched and the new pixel **p**_1_ is less than the set arm length *L*_1_.
(3) When the arm length *D*_s_ (**p**_1_, **p**) of the point to be matched and the new pixel **p**_1_ is greater than the set arm length *L*_2_, but less than the set arm length *L*_1_, and the gray difference between **p**_1_ and **p** is less than the smaller threshold *τ*_2_.

The window growth rules are the same for the vertical orientation as for the horizontal orientation.

On the one hand, this can better find areas with small differences in gray values and limit the difference in growth to the next point, avoiding the window exceeding the image boundary. On the other hand, it ensures that larger windows are available in areas with weak textures, and areas of the right size are available in areas rich in texture.

When the cross-based window of pixel **p** is constructed, a cost aggregation is per formed. The aggregate generation value of pixel **p** can be obtained by accumulating the pixel generation value in the horizontal direction, adding this value to the vertical direction, and then accumulating the pixel generation value in the vertical direction.

In order to effectively reduce the mismatching rate in the depth discontinuities, four iterations are used in the cost aggregation. The first and third iterations grow the window first along the horizontal direction, and then along the vertical direction. The second and fourth iterations grow in the opposite direction. The smaller of the four iterations is taken as the cost aggregation value.

## 4. Experiment

The algorithms were written using the C++-OpenCV environment in the VisualStudio2017 development platform. The test hardware device was a CPU with 8G of RAM, and the operating system was Windows.

### 4.1. Validation Analysis of the I-SGM Algorithm

**Figure 4:**
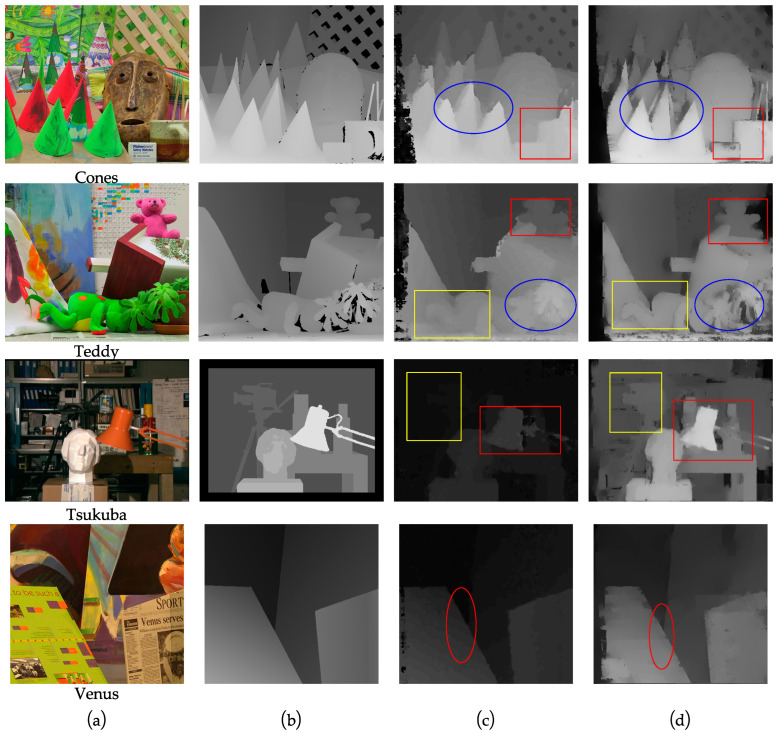
Comparison of algorithm results. (**a**) The original diagram corresponding to the four groups of images of Cones, Teddy, Tsukuba and Venus, respectively; (**b**) the standard disparity map corresponding to the four groups of images; (**c**) the disparity map of the SGM algorithm corresponding to the four groups of images; (**d**) the disparity map of the I-SGM algorithm corresponding to the four groups of images. The rectangular boxes in the figure are there to make the contrast clear.

In order to verify the effectiveness of the I-SGM algorithm in this paper, experiments were carried out on pairs of images from existing datasets. Four sets of standard test images of Cones, Teddy, Tsukuba and Venus [23,42] were used. The experimental results are shown in Figure 4. Figure 4a shows the original left image of the four standard image pairs; Figure 4b shows the standard disparity map of the corresponding image; Figure 4c shows the disparity map obtained using the SGM algorithm; and Figure 4d shows the disparity map obtained using the I-SGM algorithm.

When comparing Figure 4c,d with the standard disparity map in Figure 4b, it can be seen that both algorithms have certain shortcomings, but the I-SGM algorithm is superior to the SGM algorithm. From the circled area in Figure 4c, it can be seen that the disparity map generated by the SGM algorithm has unclear contours, and the disparity effect is significantly poorer. From Figure 4d, it can be seen that the disparity maps generated by the I-SGM algorithm have clearer and more complete contours, with better continuity of grey levels at the edges of the image, and are closer to the standard disparity maps.

In order to further quantitatively verify the effectiveness of the I-SGM algorithm, the SAD algorithm, the SGM algorithm, and the I-SGM algorithm are compared and tested. Additionally, the matching speed and accuracy of each algorithm are compared in Table 1.

As can be seen from Table 1, the SAD algorithm requires the least running time, but the lowest matching accuracy. The I-SGM algorithm is superior to the SGM algorithm in terms of matching speed and accuracy. The data in Table 1 show that the I-SGM algorithm reduces the running time by an average of 1.41 s and increases the matching accuracy by an average of 6.12% compared to the SGM algorithm.

**Figure 5:**
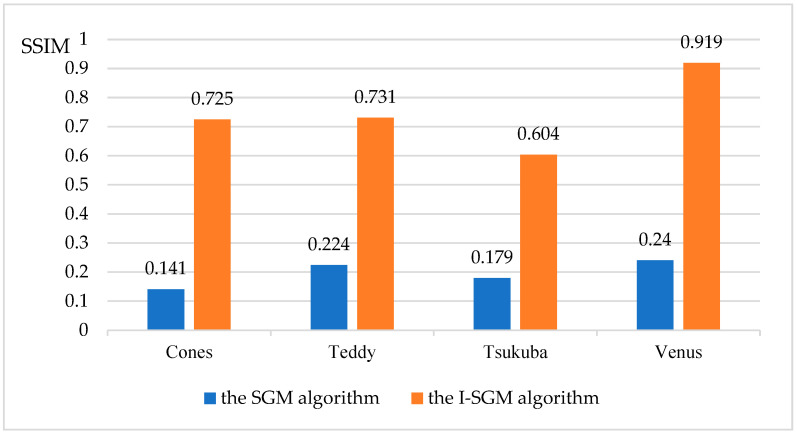
Comparison of the SSIM for algorithms.

**Figure 6:**
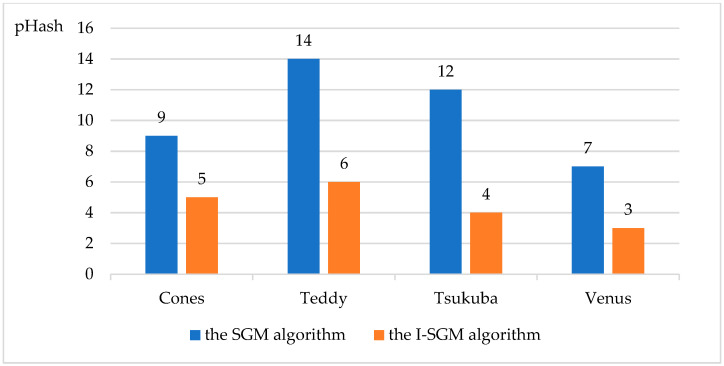
Comparison of the pHash for algorithms.

**Figure 7:**
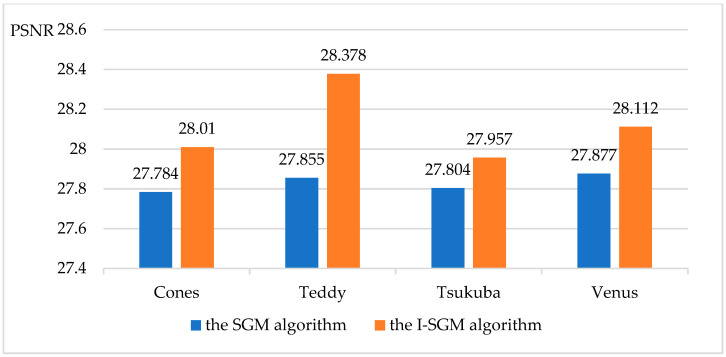
Comparison of the PSNR for algorithms.

**Figure 8:**
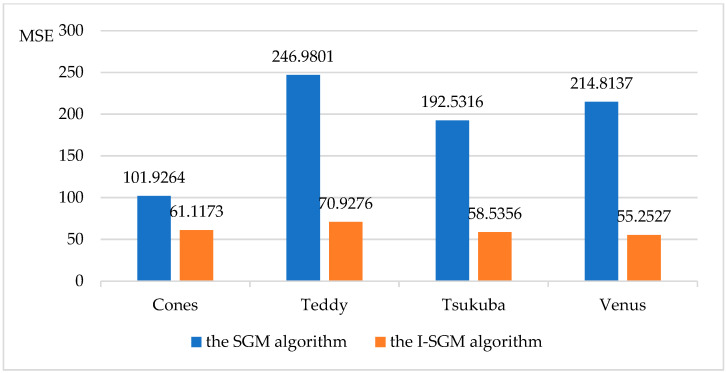
Comparison of the MSE for algorithms.

This paper also compares the objective performance metrics (SSIM, Perceptual Hash, PSNR, MSE) of the SGM algorithm and the I-SGM algorithm, as shown in Figure 5, Figure 6, Figure 7 and Figure 8.

From Figure 5 and Figure 6, it can be seen that the I-SGM algorithm has a higher Structure Similarity Index Measure (SSIM) than the SGM algorithm, while the Hamming distance value obtained using the Perceptual Hash (pHash) algorithm is smaller than that of the SGM algorithm. This indicates that the disparity maps obtained using the I-SGM algorithm are more similar to the standard maps. In Figure 7, the Peak Signal-to-Noise Ratio (PNSR) of the SGM algorithm is significantly lower than that of the I-SGM algorithm. This indicates that the I-SGM algorithm obtains disparity maps with less noise and is able to improve noise immunity. As can be seen in Figure 8, the Mean Square Error (MSE) of the SGM algorithm is much larger than that of the I-SGM algorithm. This result indicates that the quality of the disparity maps obtained by the I-SGM algorithm is better than that of the SGM algorithm.

### 4.2. Analysis of Noise Immunity

**Figure 9:**
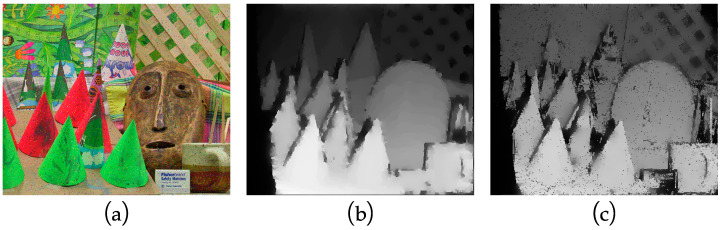
Comparison charts with the addition of noise (take the image cones with 2% added noise as an example). (**a**) Original image with 2% noise added; (**b**) The disparity map of the SGM algorithm; (**c**) The disparity map of the I-SGM algorithm.

To further verify the robustness of the I-SGM algorithm against noise, the disparity map was analyzed for noise immunity. The algorithm was compared with the SGM algorithm. Salt and pepper noise was added to the test image cones, with noise densities of 2%, 4%, 6% and 8%, respectively. The disparity maps are shown in Figure 9. The matching rates are shown in Table 2.

From Figure 9 and Table 2, it can be seen that the matching effect of the I-SGM algorithm is better than that of the SGM algorithm under a noise density of 0–8%, and the matching rate is higher. The matching rate of I-SGM algorithm fluctuates less under the influence of less noise, while the matching rate of the SGM algorithm is slightly different. With the increase in noise density, the matching rate of the disparity map under different algorithms decreases, but the decrease in the I-SGM algorithm is less than that of SGM algorithm, which shows that the I-SGM algorithm has better robustness against the influence of noise.

### 4.3. Experiments in a Simulated Lunar Surface Environment

**Figure 10:**
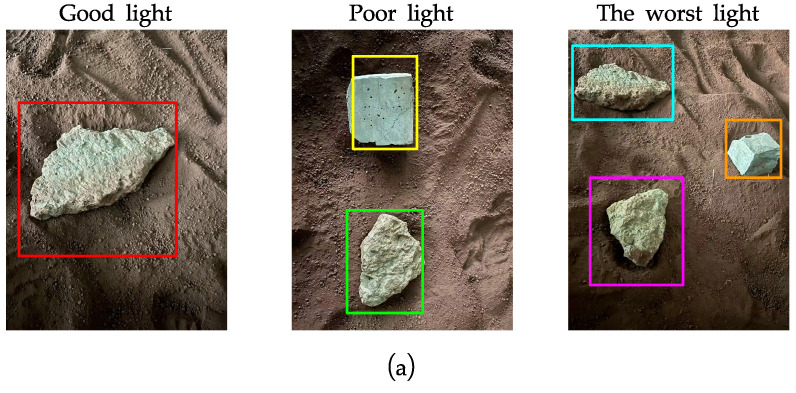

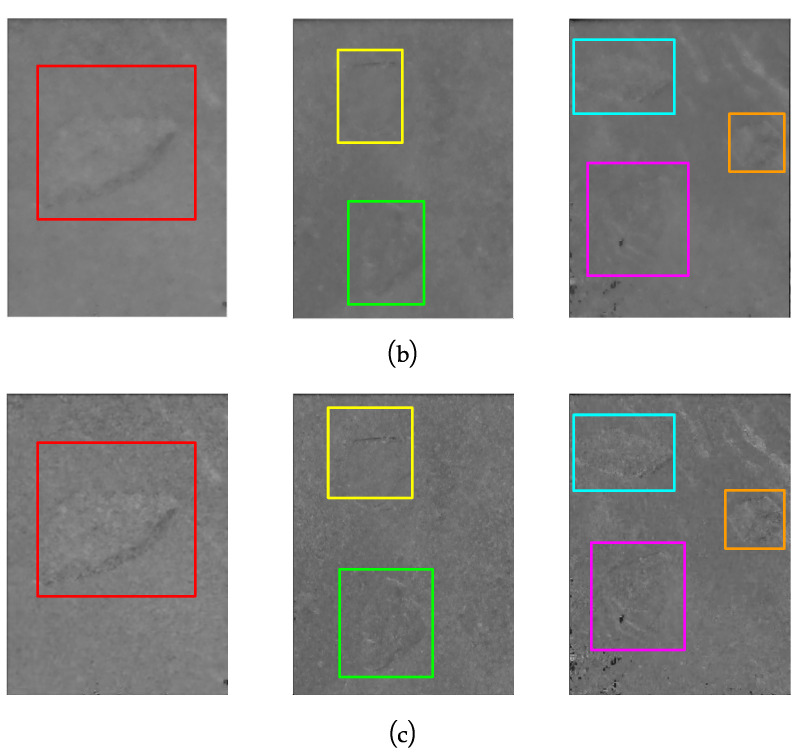
Experimental diagram of the simulated lunar surface environment. (**a**) Original maps of the different simulated environments; (**b**) disparity maps of the SGM algorithm in the different simulated environment; (**c**) disparity maps of the I-SGM algorithm in the different simulated environment. The rectangular boxes in the figure are there to make the contrast clear.

The I-SGM algorithm is implemented based on a lunar surface environment, so the experiment was carried out in the simulated Lunar Experiment Module at Southeast University to verify the practical feasibility of the algorithm. In the Lunar Experiment Module, simulated lunar soil was used to pave the floor of the module, and stones were used to simulate moon rocks. The model for simulating lunar soil is CAS-1, which is developed and produced by the Chinese Academy of Sciences. The material composition and mechanical properties of the simulant are similar to those of the lunar soil sampled from the Apollo 14 landing site [43]. The images in the simulated environment acquired using the binocular camera are shown in Figure 10a (exemplified by the left image). The obtained disparity maps are shown in Figure 10b,c. From Figure 10b,c, it can be seen that both the SGM algorithm and I-SGM algorithm can display the contours of obstacles, but it is obvious that the contours of obstacles are clearer in the disparity map obtained using the I-SGM algorithm. As the light becomes weaker, the edges of the obstacle contour can still be clearly seen in the disparity map obtained using the I-SGM algorithm, while the contour of the obstacle is not clear in the disparity map obtained using the SGM algorithm.

## 5. Conclusions

In summary, this paper focuses on the SGM algorithm and proposes the I-SGM algorithm to address the problems of low matching accuracy and weak noise immunity in the SGM algorithm. Firstly, the Census algorithm, which has more robustness to illumination changes, is used in cost calculation to replace the original mutual information-based cost calculation. Meanwhile, the Census algorithm is improved by re-determining the reference pixel value, which improves the problem of the original algorithm relying too much on the central pixel value, effectively reduces the influence of noise on the algorithm, and improves the noise immunity. Secondly, in order to solve the problem of the fixed rectangular window of the original Census algorithm affecting its matching rate, an improved idea of an adaptive window based on the data of connected components is proposed, which can reduce the running time of the algorithm. Finally, Cross-based cost aggregation in the AD-Census algorithm is used in cost aggregation to improve the accuracy of the algorithm.

Algorithm validation experiments were conducted on the standard test image pairs provided by the Middleburry website, and comparative analysis revealed that, compared to the SGM algorithm, the I-SGM algorithm reduced the execution time by an average of 1.41 s and increased matching accuracy by an average of 6.12% for all examined image sets. This paper also validates the noise immunity of the I-SGM algorithm and compares it with the SGM algorithm. Under the interference of salt and pepper noise, the matching effect of the I-SGM algorithm is significantly better than that of the SGM algorithm. As the noise density increases, the matching rate of the I-SGM algorithm also decreases less significantly than that of the SGM algorithm. The experimental results demonstrate that the I-SGM algorithm can improve matching speed and accuracy, effectively reduce the effects of noise on images, and achieve superior matching outcomes. By further applying the I-SGM algorithm to the simulated lunar environment, it is found that the I-SGM algorithm can better help lunar exploration equipment detect obstacles encountered while travelling on the lunar surface, which has a complex terrain and dim light. The I-SGM algorithm improved matching accuracy, but the improvement in computation time was not very satisfactory. In future studies, deep learning techniques can be introduced and combined with the stereo matching algorithm to train a suitable model to improve matching efficiency.

## Figures and Tables

**Table 1:** Comparison of algorithm performance.

Image Title	SAD Algorithm		SGM Algorithm		I-SGM Algorithm	
	Matching Time/s	Matching Rate/%	Matching Time/s	Matching Rate/%	Matching Time/s	Matching Rate/%
Cones	30.13	73.14	60.42	86.68	58.63	90.25
Teddy	30.59	74.46	61.36	86.31	60.29	91.72
Tsukuba	28.60	80.15	45.61	87.64	44.92	96.33
Venus	25.96	83.52	44.94	88.47	42.85	95.28

**Table 2:** Matching rate of two algorithms with different noise densities.

Noise Density	Matching Rate/%	
	SGM Algorithm	I-SGM Algorithm
0	86.68	90.25
2%	85.74	89.58
4%	83.99	89.03
6%	82.15	88.24
8%	80.87	87.51

## Data Availability

Publicly available datasets were analyzed in this study. This data can be found here: [vision.middlebury.edu].

## References

[1] Tang H., Zhu H., Tao H., Xie C. (2022). An Improved Algorithm for Low-Light Image Enhancement Based on RetinexNet. Appl. Sci..

[2] Huang H., Tao H., Wang H. A Convolutional Neural Network Based Method for Low-Illumination Image Enhancement. Proceedings of the 2nd International Conference on Artificial Intelligence and Pattern Recognition.

[3] Liu X., Li Q., Xu Y., Wei X. (2022). Point Cloud Intensity Correction for 2D LiDAR Mobile Laser Scanning. Wirel. Commun. Mob. Comput..

[4] Wang Y., Gu M., Zhu Y., Chen G., Xu Z., Guo Y. (2022). Improvement of AD-Census Algorithm Based on Stereo Vision. Sensors.

[5] Li J., Hu S., Li Q., Chen J., Leung V.C., Song H. (2020). Global Visual and Semantic Observations for Outdoor Robot Localization. IEEE Trans. Netw. Sci. Eng..

[6] Mur-Artal R., Tardós J.D. (2017). Orb-Slam2: An Open-Source Slam System for Monocular, Stereo, and Rgb-d Cameras. IEEE Trans. Robot..

[7] Chen J., Xie F., Huang L., Yang J., Liu X., Shi J. (2022). A Robot Pose Estimation Optimized Visual SLAM Algorithm Based on CO-HDC Instance Segmentation Network for Dynamic Scenes. Remote Sens..

[8] Roberts L.G. (1963). Machine Perception of Three-Dimensional Solids. Ph.D. Thesis.

[9] Marr D., Poggio T. (1979). A Computational Theory of Human Stereo Vision. Proc. R. Soc. Lond. B Biol. Sci..

[10] Zhao L., Liu Y., Men C., Men Y. (2022). Double Propagation Stereo Matching for Urban 3-D Reconstruction From Satellite Imagery. IEEE Trans. Geosci. Remote Sens..

[11] Hamzah R.A., Kadmin A.F., Hamid M.S., Ghani S.F.A., Ibrahim H. (2018). Improvement of Stereo Matching Algorithm for 3D Surface Reconstruction. Signal Process. Image Commun..

[12] Mei J., Yang X., Wang Z., Chen X., Xi J. (2021). A Topology-Based Stereo Matching Method for One Shot 3D Measurement Using Coded Spot-Array Structured Light. Sensors.

[13] Mercorelli P. (2017). A Fault Detection and Data Reconciliation Algorithm in Technical Processes with the Help of Haar Wavelets Packets. Algorithms.

[14] Wang T., Sun Y. Fast Stereo Matching Method Based on Two-Step AD-Census Fusion. Proceedings of the 2021 International Conference of Optical Imaging and Measurement (ICOIM).

[15] Rukhsar L., Bangyal W.H., Nisar K., Nisar S. (2022). Prediction of Insurance Fraud Detection Using Machine Learning Algorithms. Mehran Univ. Res. J. Eng. Technol..

[16] Menze M., Geiger A. Object Scene Flow for Autonomous Vehicles. Proceedings of the 2015 IEEE Conference on Computer Vision and Pattern Recognition (CVPR).

[17] Xue H., Huynh D.Q., Reynolds M. Pedestrian Tracking and Stereo Matching of Tracklets for Autonomous Vehicles. Proceedings of the 2019 IEEE 89th Vehicular Technology Conference (VTC2019-Spring).

[18] Torresani A., Menna F., Battisti R., Remondino F. (2021). A V-SLAM Guided and Portable System for Photogrammetric Applications. Remote Sens..

[19] Sumetheeprasit B., Rosales Martinez R., Paul H., Ladig R., Shimonomura K. (2023). Variable Baseline and Flexible Configuration Stereo Vision Using Two Aerial Robots. Sensors.

[20] Ma W.-P., Li W.-X., Cao P.-X. (2020). Binocular Vision Object Positioning Method for Robots Based on Coarse-Fine Stereo Matching. Int. J. Autom. Comput..

[21] Bangyal W., Jamil A., Abbas Q. (2013). Recognition of Off-Line Isolated Handwritten Character Using Counter Propagation Network. Int. J. Eng. Technol..

[22] Ji W., Meng X., Qian Z., Xu B., Zhao D. (2017). Branch Localization Method Based on the Skeleton Feature Extraction and Stereo Matching for Apple Harvesting Robot. Int. J. Adv. Robot. Syst..

[23] Scharstein D., Szeliski R. (2002). A Taxonomy and Evaluation of Dense Two-Frame Stereo Correspondence Algorithms. Int. J. Comput. Vis..

[24] Hirschmuller H. (2007). Stereo Processing by Semiglobal Matching and Mutual Information. IEEE Trans. Pattern Anal. Mach. Intell..

[25] Guo S., Xu P., Zheng Y. (2016). Semi-Global Matching Based Disparity Estimate Using Fast Census Transform. Proceedings of the 2016 9th International Congress on Image and Signal Processing, BioMedical Engineering and Informatics (CISP-BMEI).

[26] Zhang X., Dai H., Sun H., Zheng N. (2020). Algorithm and VLSI Architecture Co-Design on Efficient Semi-Global Stereo Matching. IEEE Trans. Circuits Syst. Video Technol..

[27] Pan X., Jun G., Xu Y., Xu Z., Li T., Huang J., Qiao W. (2021). Improved Census Transform Method for Semi-Global Matching Algorithm. Proceedings of the 2021 26th International Conference on Automation and Computing (ICAC).

[28] Zhang L., Cai F., Wang J., Lv C., Liu W., Guo G., Liu H. (2022). The SGM Algorithm Based on Census Transform for Binocular Stereo Vision. Proceedings of the 2022 International Conference on Machine Learning and Knowledge Engineering (MLKE).

[29] Liu J., He H., Nie Y., Wang J. RS-RSGM: A Revised Semi-Global Matching for Remote Sensing Image. Proceedings of the International Conference on Computer Vision, Application, and Design (CVAD 2021).

[30] Bu P., Zhao H., Yan J., Jin Y. (2021). Collaborative Semi-Global Stereo Matching. Appl. Opt..

[31] Deng C., Liu D., Zhang H., Li J., Shi B. (2023). Semi-Global Stereo Matching Algorithm Based on Multi-Scale Information Fusion. Appl. Sci..

[32] Sawant P., Temburu Y., Datar M., Ahmed I., Shriniwas V., Patkar S. (2020). Single Storage Semi-Global Matching for Real Time Depth Processing. Proceedings of the Computer Vision, Pattern Recognition, Image Processing, and Graphics: 7th National Conference, NCVPRIPG 2019.

[33] Jiao J., Yang Q., He S., Gu S., Zhang L., Lau R.W.H. (2017). Joint Image Denoising and Disparity Estimation via Stereo Structure PCA and Noise-Tolerant Cost. Int. J. Comput. Vis..

[34] Schimmack M., Mercorelli P. (2019). An Adaptive Derivative Estimator for Fault-Detection Using a Dynamic System with a Suboptimal Parameter. Algorithms.

[35] Fu L., Peng G., Song W. (2016). Histogram-Based Cost Aggregation Strategy with Joint Bilateral Filtering for Stereo Matching. IET Comput. Vis..

[36] Gan Y., Hamzah R.A., Anwar N.S.N. Local Stereo Matching Algorithm Based on Pixel Difference Adjustment, Minimum Spanning Tree and Weighted Median Filter. Proceedings of the 2018 IEEE Conference on Systems, Process and Control (ICSPC).

[37] Viola P., Wells W.M. (1995). Alignment by Maximization of Mutual Information. Proc. IEEE Int. Conf. Comput. Vis..

[38] Jodoin P.-M., Mignotte M. (2004). An Energy-Based Framework Using Global Spatial Constraints for the Stereo Correspondence Problem. Proceedings of the 2004 International Conference on Image Processing, 2004. ICIP’04.

[39] Zinner C., Humenberger M., Ambrosch K., Kubinger W. (2008). An Optimized Software-Based Implementation of a Census-Based Stereo Matching Algorithm. Proceedings of the Advances in Visual Computing: 4th International Symposium, ISVC 2008.

[40] Xinjun P., Jun H., Yong T., Yuzhi S., Yuji Y. (2017). Anti-Noise Stereo Matching Algorithm Based on Improved Census Transform and Outlier Elimination. Acta Opt. Sin..

[41] Mei X., Sun X., Zhou M., Jiao S., Wang H., Zhang X. (2011). On Building an Accurate Stereo Matching System on Graphics Hardware. Proceedings of the 2011 IEEE International Conference on Computer Vision Workshops (ICCV Workshops).

[42] Scharstein D., Szeliski R. High-Accuracy Stereo Depth Maps Using Structured Light. Proceedings of the 2003 IEEE Computer Society Conference on Computer Vision and Pattern Recognition.

[43] Chen Z.-H., Xu Z.-D., Lu H.-F., Yu D.-Y., Yang J.-Z., Pan B., Zhao X.-L., Hu Z.-W., Zhen S.-C. (2023). Novel Robust Control Strategy for the Mechanical Legs of Lunar-Based Equipment. J. Aerosp. Eng..

